# HDRPS+: a new affective pictorial scale applicable to organizational contexts

**DOI:** 10.3389/fpsyg.2025.1498143

**Published:** 2025-08-08

**Authors:** Ping Liu, Ya’Nan Wang, Yanlin Liu, Jiangning Hu, Yunyi Li, Ke Zhao, Jian’guo Mao

**Affiliations:** ^1^Business School, Sichuan University, Chengdu, China; ^2^School of Economics and Management, Tibet University, Lhasa, China; ^3^School of the Art Institute of Chicago, Chicago, IL, United States; ^4^College of Electronics and Information Engineering, Sichuan University, Chengdu, China

**Keywords:** HDRPS+, affective measurement, organizational contexts, pictorial scale, valence, arousal

## Abstract

**Introduction:**

Repeatedly capturing individuals’ emotions is challenging in organizational settings, especially for low-literacy groups, and existing pictorial scales cover arousal only narrowly. We therefore developed the Highly Dynamic and Reusable Picture-based Scale Plus (HDRPS+), an optimized successor to HDRPS that measures valence and arousal simultaneously.

**Methods:**

Three sub-studies were conducted. (1) Picture pool construction: 20 thematic images were created to span the affective space. (2) Picture screening: crowdsourced ratings anchored each image’s valence -arousal coordinates. (3) Validation: a 7-day diary study with 442 participants (age 13-69, M = 27.06) tested reliability and validity.

**Results:**

HDRPS+ achieved good user retention, with 80.3 % of participants providing data on at least five days. It also showed acceptable stability (consistency = 0.69 valence, 0.65 arousal) without materially influencing the affect it is intended to measure. Correlations with the Self-Assessment Manikin (SAM) confirmed concurrent validity (r = 0.63 for valence; 0.52 for arousal), while all coefficients with PANAS were < 0.45, supporting discriminant validity. Participants judged the scale accurate or very accurate in 78 % of cases, and indirect checks (vs. SAM) indicated reduced social_desirability bias.

**Discussion:**

HDRPS+ is low_cost, quick, and well_tolerated, enabling continuous affect tracking in diverse organizational settings. Future work should keep refining emotional granularity, broaden application formats, and test cross_cultural use. HDRPS+ images with normative scores are available at https://osf.io/d4wcn.

## 1 Introduction

Affect (or emotion) is a critical factor influencing prosocial behavior (Eisenberg, 2020), innovation (Davis, 2009; Park et al., 2022), safety attention (Wang and Liao, 2021), and performance (Inness et al., 2010; Lee et al., 2017) of organizational members. Accurately identifying and coping with affects of organizational members is essential for improving organizational competitiveness and adaptability.

Currently, there are relatively few affect measurement methods applicable to organizational contexts. Affects are dynamic and highly variable (Wang and Liao, 2021; Weiss and Cropanzano, 1996), yet organizational behavior research often relies on lengthy questionnaires administered at the start and end of studies to minimize disruption to subjects’ daily life (Pollak et al., 2011). Such methods, while informative, may not capture the dynamic nature of affects due to reliance on recall, raising concerns about the influence of autobiographical memory, mood congruence (Isomursu et al., 2007; Wilhelm and Schoebi, 2007), and recency effects (Pollak et al., 2011; Wissmath et al., 2010) on the results.

Recognizing these constraints, the remainder of the Introduction first surveys contemporary approaches to ambulatory affect assessment, from wearable physiological sensors to pictorial self-report instruments, and then evaluates their limitations when rapid, low-literacy tracking of both valence and arousal is required in workplace settings. This analysis highlights an enduring need for a lightweight tool that can repeatedly capture both dimensions among diverse employees—a need addressed by the HDRPS+ introduced in this study.

### 1.1 Ambulatory assessment of affect

In organizational settings, employees’ affective states are highly dynamic. Affective Events Theory asserts that discrete daily work incidents trigger short-lived emotional reactions that subsequently color attitudes and behavior (Weiss and Cropanzano, 1996). Diary and experience-sampling research confirms this volatility: a substantial share of the total variance in momentary positive and negative affect resides within rather than between persons (Ilies et al., 2015). Such intra-individual fluctuations matter: ambulatory assessment studies show that transient shifts in affect forecast on-the-spot safety compliance, vigilance errors and interaction quality (Klumb et al., 2009). Therefore, organizations require assessment tools that can capture affect *in situ* and at cadences fast enough to support just-in-time interventions, for example, prompting micro-breaks when arousal drops or alerting supervisors when tension spikes.

Ambulatory assessment therefore seeks to record psychological states in situ through computer-assisted self-reports, behavioral logs or physiological sensors while people perform their normal duties (Bachmann et al., 2015). Likewise, ambulatory assessment is highly suitable for affective research, which are complex neurobiological and psychological phenomena characterized by high variability (Weiss and Cropanzano, 1996).

Three broad methodological streams can be distinguished: Physiological indicator measures, External behavioral observations, and Self-reports. (1) Physiological measurements monitor heart-rate variability, electromyography, skin conductance, and other autonomic or central nervous system signals. This approach yields comprehensive data but remains intrusive and can disrupt normal activity (Cowie and Douglas-Cowie, 1996). Even though recent research has introduced the feasibility of lightweight devices such as smartwatches (Toshnazarov et al., 2024), detailed physiological data continue to pose non-trivial privacy compliance risks, and the costs of both hardware and software still remain substantial (Bolpagni et al., 2024; Doherty et al., 2025). (2) External behavioral observations focus on visible cues like facial expressions, postures, and voice tone. Utilizing high-definition cameras and streaming video for real-time analysis, this approach provides a relatively objective measure of emotional fluctuations. However, this non-intrusive method could infringe on personal privacy and confidentiality, making it less viable in organizational settings (Betella and Verschure, 2016). (3) In the self-reporting method, emotion is typically assessed through textual scales. Unfortunately, although such methods collect extensive emotional data, it requires that the scales be simple and engaging, and may pose significant challenges for individuals with limited reading skills (Cranford et al., 2006; Ebner-Priemer and Sawitzki, 2007; Wilhelm and Schoebi, 2007).

Taken together, existing ambulatory techniques struggle to balance ecological validity, intrusiveness and scalability in organizational contexts. Physiological measures and external behavioral observations are costly and primarily limited to experimental scenarios. Textual self-report methods, though cheaper and more versatile, require well-designed scales and a certain level of participant literacy. Considering these limitations, pictorial scales may prove to be a solution.

### 1.2 Pictorial scales

Pictorial scales are a measurement method that utilizes visual elements to convey the meaning of items (Sauer et al., 2021). These scales offer several advantages, including simplicity (Wissmath et al., 2010), rapidity (Schreiber and Jenny, 2020), low dropout rates (Baumgartner et al., 2019), and low cognitive demands (Desmet et al., 2016; Obaid et al., 2015). These scales are particularly effective for individuals with lower educational attainment or limited reading skills. Consequently, pictorial scales can be better suited to organizational settings that include a diverse workforce, especially employees with limited literacy (Sauer et al., 2021).

Depending on different forms of presentation, pictorial scales can be divided into three categories: grid-based, humanoid-based, and realistic-picture-based, as shown in Table 1. As for grid-based scales, some typically use a two-dimensional affective space (valence * arousal), such as Affect Grid (Russell et al., 1989) and Feeltrace (Cowie et al., 2000). However, grid-based tools also have notable drawbacks. Because valence and arousal are reported with a single click inside a matrix, respondents must trade one dimension against the other, which lowers the effective resolution for each construct. Locating a precise cell further imposes a non-trivial cognitive load that slows response time and disadvantages participants with limited numeracy or literacy (Cowie and Douglas-Cowie, 1996; Russell et al., 1989).

**Table 1:** An overview of several typical pictorial scales.

Scale	Source	Display format	Preview	Measured dimensions
Affect grid	Russell et al. (1989)	Grid	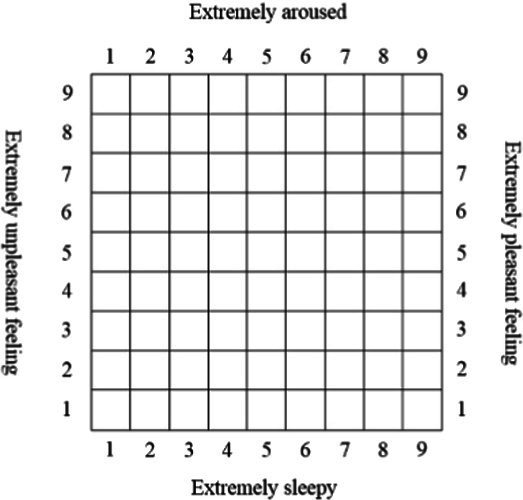	Pleasure and arousal
Feeltrace	Cowie et al. (2000)	Grid	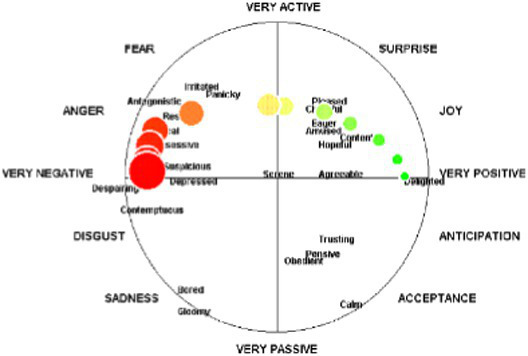	Activation and positivity
SAM	Bradley and Lang (1994)	Humanoid	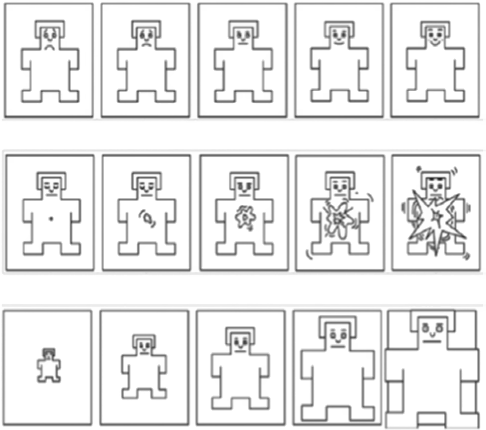	Pleasure, arousal, and dominance
Affective slider	Betella and Verschure (2016)	Humanoid	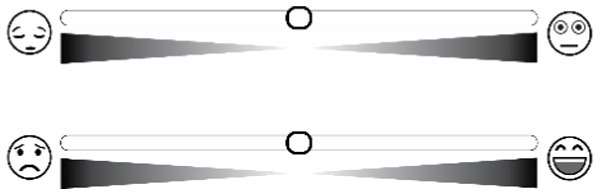	Pleasure and arousal
PAM	Pollak et al. (2011)	Realistic pictures	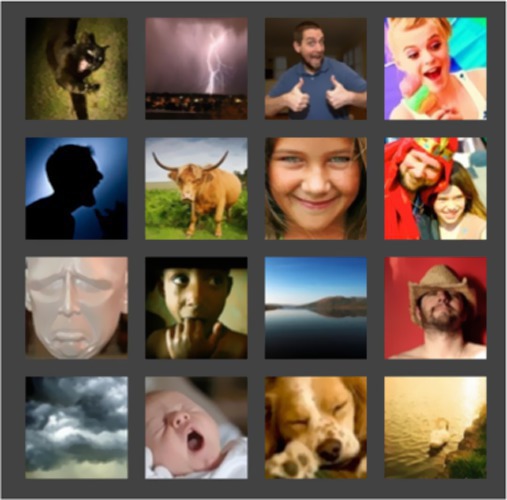	Valence and arousal
HDRPS	Liu et al. (2023)	Realistic pictures	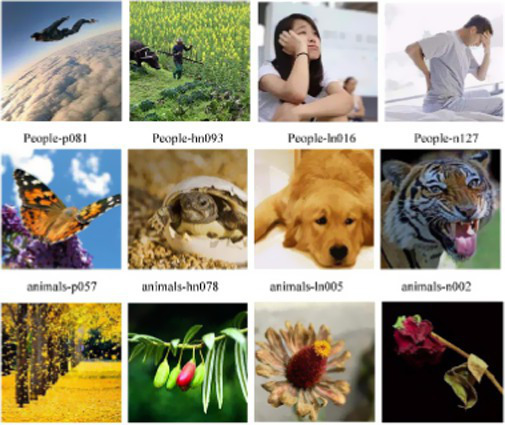	Valence

Humanoid pictorial scales utilize emoticons or humanoid cartoons as anchors. For instance, the Self-Assessment Manikin (SAM) uses five progressively changing humanoid cartoons to measure valence, arousal, and dominance. Humanoid or manikin scales likewise present limitations. Interpretation of the stylized figures is filtered through culturally embedded gender and age stereotypes, leading to systematic bias across demographic groups. Moreover, the extreme postures used to depict very high arousal become visually ambiguous, reducing discriminability at the upper end of the arousal continuum (Betella and Verschure, 2016; Bradley and Lang, 1994).

The realistic-picture-based scales are based on psychological projection techniques and use a multitude of real pictures as anchors, measuring emotions by having participants select the image that best matches their mood. For example, the Photographic Affect Meter (PAM) scale presents 16 images of different categories and themes in a single session, and determines participant’s valence and arousal based on affective labels corresponding to the selected image (Pollak et al., 2011). Similarly, the Highly Dynamic and Reusable Picture-based Scale (HDRPS) displays a quartet of thematically matched photographs (e.g., four animal images) that span an unpleasant-to-pleasant continuum. Participants click the single image that best matches their current affect, and its ordinal rank is recorded as the valence score for that trial (Liu et al., 2023).

Compared with instruments like grid and manikin, realistic photographs offer three distinct advantages for high-frequency organizational use. First, they deliver rich, multimodal cues that can be decoded in under a few seconds, matching the pace of diary sampling (Lang et al., 2005). Second, the concrete imagery eliminates the abstraction burden of coordinate mapping or icon interpretation, thereby reducing error variance and respondent fatigue (Dan-Glauser and Scherer, 2011). Third, photo sets can portray diverse contexts and actor identities, which helps minimize gender- or culture-specific stereotype bias and yields smaller social-desirability effects than stylized faces (Betella and Verschure, 2016). These properties make realistic-picture scales particularly suitable for continuous affect monitoring in the workplace.

As the main problem addressed in this study is the continuous measurement of individual affects in organizational contexts. On the one hand, in labor-intensive sectors such as construction, manufacturing, warehousing, and hospitality where many frontline employees have limited reading proficiency, measurement tools must remain simple and easy to understand. On the other hand, considering the need for repeated measurements, these tools should also be engaged to enhance participant motivation. Considering these factors, measurement tools that use real images as anchors are particularly suitable for this context.

However, there are relatively few realistic-picture-based scales available, and those that exist exhibit obvious limitations. For example, the PAM presents 16 images simultaneously to measure emotional states, raising unresolved questions about whether this multi-image display method might alter the subjects’ emotional states. Second, the image materials in PAM have not undergone rigorous rating, making the use of self-reported emotions as emotional labels for the images less precise. Third, PAM also mixes images of different types and themes, which does not account for the influence of personal preferences or visual appeal on the measurement results (Sauer et al., 2021). Finally, with only 100 images in its database and presenting 16 images at once, there is a high possibility of repetition, which can decrease response motivation.

### 1.3 Contributions and limitations of HDRPS

Considering the limitations of existing realistic-picture-based scales, our team previously developed HDRPS. This scale initially collected 22,054 raw images, which were subjected to a three-step evaluation process: image usability testing, emotional type assessment, and emotional scoring experiments. Through more rigorous evaluations, images were carefully selected and categorized, resulting in the creation of a structured pictorial scale with 3,386 images, each tagged with detailed attribute labels.

Thus, HDRPS refines the selection and display of images to enhance measurement accuracy and reduce bias. By presenting fewer images at once and employing a rigorous vetting process for each image, it ensures more consistent and reliable emotional assessments. Additionally, HDRPS expands the image database, thereby reducing repetition and sustaining participant engagement throughout the study. This results in a more robust and effective tool for capturing nuanced emotional responses in research settings.

However, HDRPS still faces two significant limitations that necessitate further optimization. Firstly, in terms of measurable dimensions, while HDRPS images are labeled with both valence and arousal, the scarcity of high arousal images has led to a design focus primarily on valence. This limits the scale’s ability to accurately capture the precise emotions of the subjects. Secondly, regarding the presentation of images, although HDRPS reduces the influence of personal preferences and visual appeal by displaying four images of the same category (e.g., all animal images) at once, it still fails to eliminate the impact of content differences (e.g., images of a cat, dog, bird, and fish) on the measurement outcomes. These issues highlight the need for further refinements in HDRPS to enhance its accuracy and applicability.

### 1.4 About this study

In this article, we present the development and validation of HDRPS+, an affect measurement instrument designed for real organizational contexts. In response to the practical needs for dynamic and continuous measurement, we conducted 3 sub-studies to develop and validate HDRPS+, which can continuously measure changes in valence and arousal of organizational members. As an important optimization of HDRPS, developed by our team previously (Liu et al., 2023), HDRPS+ incorporates arousal dimension into measurement process, which can more accurately characterize individuals’ emotional states. Compared to traditional emotion research instruments, HDRPS+ is shown to have broader applicability, lower economic costs, simpler testing procedures, higher response rates, and lower susceptibility to social desirability effects. As a result, it can provide valuable support for continuous affect measurement in organizations.

Specifically, the three sub-studies were built upon the foundational designs of PAM and HDRPS to refine and test HDRPS+ thoroughly. First, we identified image acquisition ideas based on the results of semi-structured interviews, and constructed a picture pool through picture acquisition and supplemental acquisition (Study 1). Then, we screened and constructed picture attributes by evaluations on available, affective category, and affective score (Study 2). Finally, after comparing different picture presentation methods, we determined the optimal measurement and conducted a large-scale trial to verify the reliability and validity of the newly developed tool (Study 3).

Compared with prior measurement tools, the special contributions of this study are fourfold. First, the development and validation process of HDRPS+ is more rigorous, providing a beneficial reference for the development of pictorial scales. Second, the design of HDRPS+ draws on the principles of simple-drawing scales such as SAM, emphasizing the correlation of appearance or content among each set of images, thus effectively controls the influence of personal preferences and image attractiveness on the measurement results. Third, HDRPS+ was developed based on the valence-arousal emotion model, using 3 same-themed images to measure the subjects’ valence and arousal, which can draw individual affect portraits more quickly and accurately. Fourth, the database comprises 20 thematic image sets, including animals, plants, and scenes, offering richer material and a more balanced category distribution that can accommodate a wide range of subsequent research designs.

## 2 Study 1: item generation

### 2.1 Step 1: determining the approach for picture acquisition

As realistic pictures are diverse and complex, clear clues for picture collection must be established when using them as measurement anchors. To this end, we recruited 57 students (29 males and 28 females, with an average age of 21.81) from Sichuan University who had participated in HDRPS picture ratings, and conducted semi-structured interviews in which lasted approximately 10 min each, aimed to address the following questions:

(1) Comparison of the difficulty level of evaluating different categories of pictures (valence and arousal) in HDRPS.
(2) What are the factors that influence the judgment of picture affects (valence and arousal)? Please provide examples.

These interviews yielded 8 h and 43 min of audio recordings, which were transcribed into 150,600 words. Using ATLAS.ti 9.0 software, the data underwent three stages of coding, resulting in 496 primary codes, 26 secondary codes, and 3 core propositions (overall factors, emotional dimensions, and picture categories). The key findings are as follows: (1) Personal preferences would affect the evaluation of pictures in animals, plants and scenes. To avoid this, HDRPS+ should use group pictures with the same main subject matter. For example, in the case of images featuring people, all images within a set should feature the same individual, while for animal images, all images within a set should have similar appearances. (2) The factors influencing the evaluation of valence and arousal differ. Therefore, image collection should be dimension-specific to accurately convey emotional meanings. (3) The collection strategy for specific images should be adapted according to the circumstances. Due to the poor emotional discernibility of object-related images, we have temporarily restricted the image categories to people, animals, plants, and scenes. Additionally, considering that expressions and postures can affect the affective judgment of animal-related images, we have only selected mammalian animals with clear and visible facial expressions during picture acquisition.

### 2.2 Step 2: picture acquisition

We referenced the methods utilized by HDRPS and NAPS (Nencki Affective Picture System, Marchewka et al., 2014) for picture acquisition. With the exception of some self-taken photographs, the majority of the pictures were obtained from publicly available networks. A total of 60 sets of pictures (415 pictures in all) were collected, including 4 sets of people, 17 sets of animals, 19 sets of plants, and 20 sets of scenes. Since it was difficult to collect pictures of the same person in different affective states from open networks, we attempted to use facial expression databases (e.g., the Japanese Female Facial Expression Database, Lyons, 2021) or screenshots of film and TV works as picture materials. However, due to the following four considerations, we ultimately decided to remove images of people (26 in total). Firstly, the purpose of developing these facial expression databases was to provide standard materials for inducing specific emotions. Therefore, using such images as emotional anchors is highly likely to induce affective changes in the subjects. Secondly, the performers in film and TV works are often celebrities, and the subjects’ preferences for these performers could influence the evaluation results. Thirdly, the emotional suggestiveness of people figures can be strong, and subjects are more susceptible to be influenced by social desirability during testing, leading to measurement bias. Fourthly, there are potential legal risks related to issues such as portrait rights.

### 2.3 Step 3: determine the evaluation baseline pictures

To ensure consistent evaluation baselines for each HDRPS+ image set (i.e., having representative “neutral/medium” images for each dimension), we individually evaluated the valence (positive, neutral, negative) and arousal (high, medium, low) dimensions of all 389 images. This approach enables the identification of the valence baseline image (with a neutral valence) and arousal baseline image (with a neutral arousal) for each set of images.

At the operational level, we utilized OpenCV and Visual Studio 2015 to adjust 389 original images to 512*512 pixel JPG images. In addition, to facilitate comparison of images in the same set, images of the same evaluation dimension within the set were presented simultaneously using slides. During the process, 5 master’s and doctoral students with more than 2 years of emotion research experience successively evaluated the valence and arousal dimension of each set of pictures and were given a 5-min break between the two modules.

The agreement percentage (AP) refers to the percentage of evaluators who perceive a particular image to belong to a specific emotional category out of all evaluators (Wang and Chu, 2013; as shown in equation 1). In this study, an 80% agreement percentage was used as the threshold for judgement. Eventually, a total of 23 sets of pictures, comprising 10 sets of animals, 5 sets of plants, and 8 sets of scenes, were identified to possess both the valence and arousal baselines.


(1) $${\mathrm{AP}}_{i}=\frac{{\mathrm{NE}}_{i}}{N}\times 100\%$$

Note: AP refers to the agreement percentage; i represents the emotional category of the image (including positive valence, neutral valence, negative valence, high arousal, medium arousal, and low arousal); ${\mathrm{NE}}_{i}$ denotes the number of evaluators who consider a certain picture to belong to the emotional category i; N represents the total number of evaluators.

### 2.4 Step 4: supplemental acquisition

As not every set among the 23 sets in Step 3 contains all categories under the arousal dimension (e.g., Scenes-1 lacks highly arousing images), and the number of pictures varied greatly between groups, we conducted supplementary picture acquisition. Except for two sets of images that were difficult to collect, the remaining 21 sets of images were moderately supplemented (10 images per set).

Following the supplemental acquisition, we obtained the initial pool for HDRPS+, which contains 9 sets of animals, 5 sets of plants, and 7 sets of scenes.

## 3 Study 2: picture screening and attribute construction

### 3.1 Step 1: usability evaluation

In this paper, we set up an available evaluation session of picture materials inspired by the development of textual scales. Twenty MBA students from Sichuan University participated in this experimental session, including 6 males and 14 females who confirmed that they were currently mentally and cognitively normal, without psychiatric diagnoses or use of psychotropic medications. As this phase was a small-scale professional evaluation rather than an effort to establish a population statistical sample, perfect gender balance was not strictly controlled.

To address potential dimensional contamination issues of evaluating valence and arousal simultaneously (Kurdi et al., 2017), we divided the experiment into two modules: valence evaluation and arousal evaluation. Prior to the experiment, the experimenter provided informed consent forms, instructions (including an introduction to the evaluation indexes and task descriptions), and scoring sheets. During the evaluation, participants judged the “availability” of each of the 21 picture sets—that is, whether the set met our predefined criteria of relevance, clarity, and specificity for both valence and arousal (see Table 2)—and then recorded a Yes/No response on the scoring sheet. Considering individual differences in evaluation speed, we did not impose restrictions on the playback speed of the images, but only required each subject to respond as quickly and accurately as possible based on their intuition.

**Table 2:** Available evaluation index.

Dimension	Index	Explanation
Valence	Relevance	Can this set of images reflect the definition and description of valence?
	Clarity	Is the emotional meaning of this set of images clear and unambiguous?
	Specificity	Can this set of images constitute a complete and distinguishable valence levels, rather than being overly general or vague?
Arousal	Relevance	Can this set of images reflect the definition and description of arousal?
	Clarity	Is the emotional meaning of this set of images clear and unambiguous?
	Specificity	Can this set of images constitute a complete and distinguishable arousal levels, rather than being overly general or vague?

The agreement percentage of 6 available evaluation indexes for each set of pictures was calculated according to Equation 1. Following the criteria proposed by Li (2014), sets of images were screened with a threshold of 60% agreement ratio. The results showed that a total of 15 sets of pictures had an agreement ratio higher than 60% for all 6 available indexes, including 8 sets of animals, 5 sets of plants, and 2 sets of scenes.

### 3.2 Step 2: affective category labelling

#### 3.2.1 Participants

Affective category evaluation was performed to determine the affective types (affective labels) of each picture. To ensure the scientific validity of picture labels, PhD students with relevant research backgrounds were recruited from Business School of Sichuan University for image evaluation. Participants were required to have studied psychology or other related courses, and have research experience in emotional science.

Subsequently, we conducted a qualification test to confirm the exact candidates of the participants. The qualification test consisted of three parts: Ishihara Color Blindness Test, Self-Rating Anxiety Scale (SAS) test, and Self-Rating Depression Scale (SDS) test. A SAS score lower than 50 and an SDS score lower than 53 indicated normal mental status (Zung, 1965, 1971), and passing all three tests was considered as passing the qualification test (Li et al., 2020). Eventually, a total of 12 applicants (6 males and 6 females, aged 25–33 years) passed the background check and qualification test, and received a compensation of 100 CNY after completing a 30-min experiment.

#### 3.2.2 Procedure

**Figure 1:**
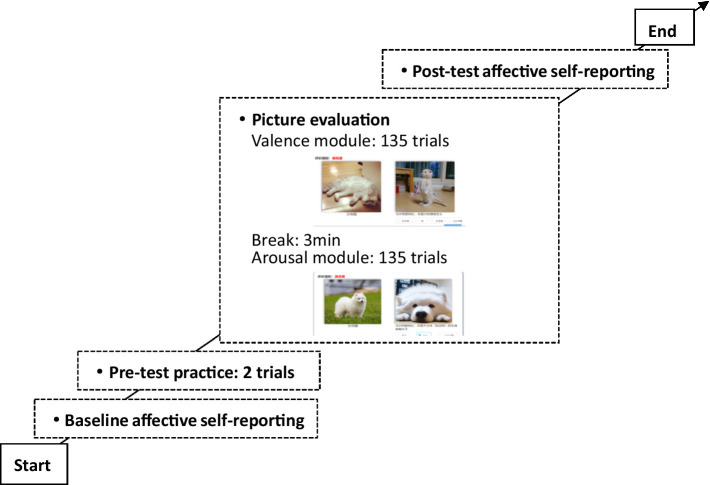
Process of affective category evaluation.

The entire experiment consisted of four phases: baseline affective self-reporting, pre-test practice, picture evaluation, and post-test affective self-reporting (as shown in Figure 1). The post-test assessment was included to verify that the rating task itself did not systematically alter participants’ mood; confirming stable pre- and post-scores allows us to attribute picture evaluations to the images rather than task-induced state changes.

We developed a web-based image-evaluation system to implement the above experiments and record all responses. Both baseline and post-test affective self-reports employed a 9-level SAM scale to distinguish individual differences in affects (Desmet et al., 2016), and the Positive and Negative Affect Schedule (PANAS) scale (Watson et al., 1988) was added to the post-test affective self-reporting session. During the picture-evaluation phase, 15 picture sets were presented in random order. Participants first completed the valence module and then the arousal module. To give each set a clear point of reference, the baseline image identified in Study 1 Step 3 was displayed on the left side of the interface, while the target image appeared on the right side of the evaluation interface. Thus, participants used a pairwise comparison method to determine the valence (options included more positive, consistent, more negative, and unable to judge) and arousal (options included higher, consistent, lower, and unable to judge) types of the images to be evaluated (Obaid et al., 2015).

#### 3.2.3 Results

Baseline self-reports indicated a mean valence of 5.92 and a mean arousal of 4.17. After the picture-evaluation task, mean valence was 6.00 and mean arousal 4.67; the mean PANAS score was 13. Paired-samples t-tests showed no significant change in valence (*p* = 0.754) or arousal (*p* = 0.438) from pre- to post-test, suggesting that participants’ emotional state remained stable during the task and therefore exerted little influence on their picture ratings. After removing redundant data due to network problems, we used the Cronbach’s alpha to calculate the consistency of the valence and arousal ratings provided by 12 subjects. It showed that Cronbach’s alpha of valence was 0.937 (Cronbach’s minimum after removal of items was 0.928), and Cronbach’s alpha of arousal was 0.929 (Cronbach’s minimum after removal of items was 0.917), which exceeded the standard set by Cook et al. (2018), and passed the reliability test.

We also calculated the valence and arousal agreement percentage of 135 evaluated pictures, respectively, based on Equation 1, and pictures with agreement percentage above 60% were retained (Li, 2014) and labeled with their corresponding emotional categories. Finally, 127 pictures were retained in the valence dimension (including 45 positive images, 27 neutral images, and 55 negative images), and 134 pictures were retained in the arousal dimension (including 48 low arousal images, 28 medium arousal images, and 58 high arousal images).

### 3.3 Step 3: affective scoring

#### 3.3.1 Method

In Step 2, the affective categories of each picture have been determined. In this section, we will further ascertain the affective score of each picture with the help of a web-based picture evaluation system to determine the accurate anchoring of pictures. To ensure the generalizability of the scoring results, this experiment did not restrict the subject’ professional backgrounds, but only tested their health status (including Ishihara Color Blindness Test, SAS test, SDS test). 79 undergraduate and graduate students from Sichuan University applied to participate in this experiment, and finally, a total of 58 subjects (23 males and 35 females; Mean of age = 22.68) formally participated in this experiment and received a reward of 30 CNY after completing the experiment. The specific operation process of this experiment is basically similar to that of Step 2, except for the 9-level scale of the baseline and post-test affective self-reporting were adjusted to a 5-level scale according to Desmet et al. (2016).

#### 3.3.2 Results

At baseline, mean valence and arousal were 3.61 and 2.88, respectively. Post-task means were 2.88 (valence) and 3.46 (arousal); the PANAS mean was 13.22. Paired-samples t-tests again revealed no significant change in valence (*p* = 0.135) or arousal (*p* = 0.669), indicating that participants’ affective state remained largely constant throughout the evaluation and had relatively small impact on their judgments. Using the Cronbach’s alpha to calculate the consistency of the 58 subjects’ picture rating results separately, and the data revealed that Cronbach’s alpha of valence was 0.991 (Cronbach’s minimum after removal of items was 0.991) and Cronbach’s alpha of arousal was 0.992 (Cronbach’s minimum after removal of items was 0.992), indicating high consistency (Li, 2014).

Based on Equations 2, 3, the agreement percentage of valence and arousal for each image were calculated, and images were selected based on a standard of 60% (Li, 2014). The results show that there were 118 remaining images in the valence dimension and 131 in the arousal dimension. Equations 4, 5 were then used to calculate the valence and arousal scores of each image.


(2) $${\mathrm{AP}}_{V}=\max(N_{1}+N_{2},N_{3},N_{4}+N_{5},N_{6})/N^{*}100\%$$

(3) $${\mathrm{AP}}_{A}=\max(M_{1}+M_{2},M_{3},M_{4}+M_{5},M_{6})/M^{*}100\%$$

Note: APV is the agreement percentage of valence; N_1_, N_2_, N_3_, N_4_, N_5_ and N_6_ are the number of people who agree that the image valence is (1) extremely negative, (2) negative, (3) the same, (4) positive, (5) extremely positive, and (6) undeterminable, respectively; N is the total number of participants in the valence evaluation; AP_A_ is the agreement percentage of arousal; M_1_, M_2_, M_3_, M_4_, M_5_ and M_6_ are the number of people who agree that the image arousal is (1) extremely low, (2) low, (3) the same, (4) high, (5) extremely high, and (6) undeterminable, respectively; M is the total number of participants in the arousal evaluation.


(4) $$S_{V}=({N_{1}}^{*}1+{N_{2}}^{*}2+{N_{3}}^{*}3+{N_{4}}^{*}4+{N_{5}}^{*}5)/(N-N_{6})$$

(5) $$S_{A}=({M_{1}}^{*}1+{M_{2}}^{*}2+{M_{3}}^{*}3+{M_{4}}^{*}4+{M_{5}}^{*}5)/(M-M_{6})$$

Note: SV is the valence score of a certain image, S_A_ is the arousal score of a certain image.

### 3.4 Step 4: picture attribute construction

Based on Equations 6, 7, we finalized the valence and arousal labels of each image, and images with consistent affective category in step 2 and step 3 were retained. A total of 115 images remained for the valence dimension, and 116 images remained for the arousal dimension. In addition, we also analyzed the data by group (see Appendix 1), and found that the remaining all 14 groups of pictures, except for Animal-8, were able to reflect the complete affective space.


(6) $$\mathrm{TV}=\{\begin{array}{l} \text{positive},\mathrm{SV}>3.5\\ \text{neutral},2.5\leq \mathrm{SV}\leq 3.5\\ \text{negative},\mathrm{SV}<2.5 \end{array}$$

(7) $$\mathrm{TA}=\{\begin{array}{l} \text{high},\mathrm{SA}>3.5\\ \text{medium},2.5\leq \mathrm{SA}\leq 3.5\\ \mathrm{low},\mathrm{SA}<2.5 \end{array}$$

Note: *Tv* is the valence category of a certain image, *T_A_* is the arousal category of a certain image.

Further analysis showed that the 14 newly collected sets were unevenly distributed—seven animal, five plant, and only two scene sets. To enrich the material and balance category counts, we supplemented the database with images from the original HDRPS database, whose pictures had already been judged using the same procedure but on a 9-point valence/arousal scale. To keep the granularity consistent across all materials, we recoded the 9-point HDRPS valence scores into five ordered categories (1–2 = very low, 3–4 = low, 5 = neutral, 6–7 = high, 8–9 = very high; see Table 3). After supplementation, the final database contained 20 thematic picture sets.

**Table 3:** Supplementary images from HDRPS.

Group	Number	Dimension	Score	Number	Dimension	Score
Animals 8	Animals-n011	Valence	1.65	/	/	/
Plants 6	Plants-p129	Valence	3.85	Plants-p107	Arousal	3.60
	Plants-hn094	Valence	3.35	Plants-ln016	Arousal	2.56
	Plants-n001	Valence	2.28	Plants-n008	Arousal	1.61
Scenes 3	Scenes-p006	Valence	3.74	Scenes-p031	Arousal	3.58
	Scenes-hn017	Valence	3.4	Scenes-hn013	Arousal	2.77
	Scenes-n011	Valence	2.21	Scenes-n010	Arousal	1.77
Scenes 4	Scenes-p340	Valence	3.98	Scenes-p293	Arousal	3.71
	Scenes-hn089	Valence	3.15	Scenes-hn116	Arousal	2.87
	Scenes-n086	Valence	2.17	Scenes-n063	Arousal	1.79
Scenes 5	Scenes-p235	Valence	3.76	Scenes-p235	Arousal	3.30
	Scenes-hn347	Valence	3.27	Scenes-hn373	Arousal	4.15
	Scenes-n234	Valence	2.08	Scenes-ln113	Arousal	1.86
Scenes 6	Scenes-p236	Valence	3.66	Scenes-p085	Arousal	3.64
	Scenes-hn366	Valence	3.18	Scenes-hn367	Arousal	2.86
	Scenes-n228	Valence	1.99	Scenes-n229	Arousal	1.69

## 4 Study 3: validation of HDRPS+

### 4.1 Step 1: presentation-mode determination

#### 4.1.1 Method

##### 4.1.1.1 Objective

In this session, we determined the optimal presentation format of the scale by comparing the accuracy rates of different presentation methods. Based on the magnitude of differences between the presented images, the presentation formats of the scale can be divided into three levels: primary, middle, and advanced (see Appendix 2). The primary-level format presented images of the same category and theme (e.g., all pictures are tigers). The middle-level format followed the testing method of HDRPS, presenting images of the same category but with multiple themes (e.g., presenting rabbits, dogs, and tigers at the same time). The advanced-level format followed the testing method of PAM, presenting images of multiple categories and themes (e.g., presenting lotus, birds, and mountains simultaneously).

##### 4.1.1.2 Participants

A total of 88 individuals applied to participate in this experiment, of whom 85 subjects (26 males, 59 females, average age 23.75) passed the Ishihara Color Blindness Test and were then randomly assigned to the experimental group (with 31 in the positive group and 29 in the negative group) and the control group (25 subjects), and each received a compensation of 10 CNY after the experiment.

##### 4.1.1.3 Procedure

**Figure 2:**
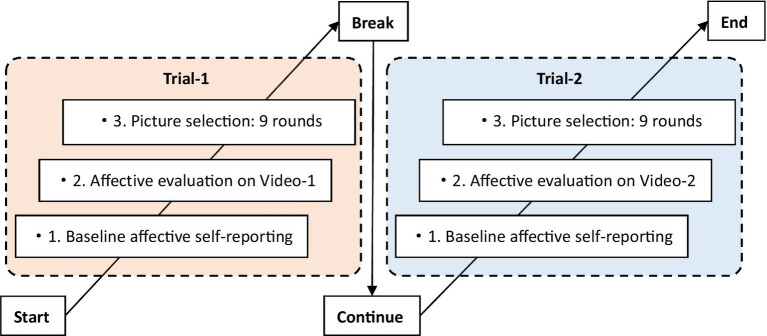
Procedure of experimental group.

The experimental group completed three phases per trial: (1) baseline affective self-reporting, (2) video affect evaluation, and (3) picture selection (see Figure 2 for details). Each trial began with the baseline affective self-reporting, where subjects were required to report their affects using the 5-point SAM scale. In the video affect evaluation phase, subjects were instructed to watch a 15-s video clip and then evaluate its valence and arousal using the SAM scale. In the picture selection phase, subjects were required to make 9 rounds of picture selection based on the emotions reflected in the videos, in terms of valence and arousal dimensions, respectively. In the valence module, the subjects were required to select 1 image from 3 images that best represented the valence level of the video; and in the arousal module, subjects were required to select 1 image from 3 images that best represented the physiological or psychological arousal level of the video. Participants were given a 60-s rest time after the first trial, after which the aforementioned processes were repeated (with new materials).

In contrast to the experimental group, the procedure in the control group merely consisted of two phases: (1) the baseline affective self-reporting, and (2) picture selection, in which the subjects were instructed to select pictures in accordance with their emotions.

##### 4.1.1.4 Performance indices

(a) Internal consistency for reliability analysis: Cronbach’s *α* for each nine-choice set; α ≥ 0.70 was deemed acceptable delineated by Cook et al. (2018). (b) Time Consumption (TC) for measurement speed analysis: Seconds from items onset to mouse click. (c) Accuracy (AC) for measurement accuracy analysis: Equations 8, 9 were used to sequentially determine the picture selection results of 85 participants for 9 rounds, while Equations 10, 11 were then employed to calculate the accuracy of HDRPS+ test results.


(8) $$\text{RVij}=\{\begin{array}{l} 1,\mathrm{Tv}=\mathrm{Tvi}\\ 0,\mathrm{Tv}\neq \mathrm{Tvi} \end{array}$$

(9) $$\text{RAij}=\{\begin{array}{l} 1,\mathrm{TA}=\mathrm{TAi}\\ 0,\mathrm{TA}\neq \mathrm{TAi} \end{array}$$

Note: *Rv_ij_* is the result of the *i*-th valence test of person *j*; *Tv* is the video valence category (experimental group) or self-rated valence category (control group); *Tv_i_* is the valence category corresponding to the *i*-th selection of pictures; *R_Aij_* is the result of the *i*-th arousal test of person j; *T_A_* is the arousal category of video (experimental group) or self-rated arousal category (control group); *T_Ai_* is the arousal category corresponding to the *i*-th selection of pictures; *i* is the order of picture selection ranging from 1 to 9, and *j* is the subject number.


(10) $$\text{ACVj}=\frac{\sum_{i=1}^{9}\text{RVij}}{9}*100\%$$

(11) $$\text{ACAj}=\frac{\sum_{i=1}^{9}\text{RAij}}{9}*100\%$$

Note: *AC_Vj_*, *AC_Aj_* are the accuracy rates of valence and arousal tests using HDRPS+ of the *j*-th individual, respectively.

#### 4.1.2 Results

##### 4.1.2.1 Reliability analysis

After deleting redundant and missing data, we obtained a total of 2,988 picture-selection results, 1,494 each for both valence and arousal dimensions. Cronbach’s α was computed across nine selection rounds for both the experimental and control groups to assess internal consistency. As shown in Table 4, the HDRPS+ demonstrates acceptable reliability as the consistency test results of picture selection were all higher than the threshold of 0.5 delineated by Cook et al. (2018).

**Table 4:** Consistency test for picture selection.

Group	Cronbach’s alpha of valence		Cronbach’s alpha of arousal	
	Original	Min after removing items	Original	Min after removing items
Experimental	0.894	0.875	0.666	0.620
Control	0.805	0.752	0.720	0.660

##### 4.1.2.2 Measurement speed analysis

The average time consumption (TC) was 8.022 s per trial, indicating quick testing and rapid judgment responses. Then, a one-way ANOVA showed a significant effect of presentation method on time consumption (*F* = 5.19, *p* < 0.001). Further comparison of means showed that TC _primary_ < TC _advanced_ < TC _middle_. Considering that image category might also influence response time, we conducted a separate analysis across categories but no significant differences emerged, although animal images were numerically the slowest to rate.

Then we analyzed the effect of demographic data such as gender and education on measurement time using an independent sample *t*-test. The results showed that gender significantly affected test speed (*F* = 9.461, *p* = 0.002), with females taking longer to select pictures.

##### 4.1.2.3 Measurement accuracy analysis

The results showed that the accuracy of the experimental group’s valence test was 65.80%, while that of the arousal test was 58.81%. The control group exhibited a valence accuracy rate of 63.56% and an arousal accuracy rate of 62.44%. Comparison of the accuracy rates of different testing methods showed that AC_primary_ > AC_middle_ > AC_advanced_. Further analysis of the measurement results for different categories of images, it was observed that plant images demonstrated the highest accuracy in valence measurement (see Table 5 for details).

**Table 5:** Accuracy of presentation methods.

Method	Experimental group		Control group	
	ACv	AC_A_	ACv	AC_A_
General	65.80%	58.81%	63.56%	62.44%
Advanced	59.48%	52.72%	59.46%	53.37%
Middle	64.94%	52.87%	63.16%	58.55%
Middle-animals	64.66%	48.28%	70%	56%
Middle-plants	68.97%	50.86%	70%	54%
Middle-scenes	61.21%	59.48%	50%	68%
Primary	72.99%	70.89%	68%	75.33%
Primary-animals	65.52%	69.83%	58%	76%
Primary-plants	76.72%	74.78%	80%	74%
Primary-scenes	76.72%	68.10%	66%	76%

Similarly, we investigated the effect of gender, education, and number of trials on accuracy using independent samples T-tests, yet the results showed that the accuracy of the HDRPS+ was not affected by these factors. We also explored the relationship between individual emotional states and measurement accuracy using correlation analysis. The data showed a significant positive correlation between valence and test accuracy (r = 0.153**), indicating that higher valences were associated with higher accuracy in HDRPS+ measurement results.

In summary, the primary mode can not only reduce the influence of personal preference and picture attractiveness on measurement results, but also has the advantages of rapid measurement and high accuracy, which is recommended as the optimal measurement method for the HDRPS+.

### 4.2 Step 2: validation based on large-scale samples

#### 4.2.1 Participants

Through Step 1, we have determined the presentation format of HDRPS+, and in this session, we would test the validity of HDRPS+ among student and working populations simultaneously. A snowball sampling technique was employed to openly recruit subjects within the community. After deleting 8 subjects due to data loss caused by network issues and 18 subjects with color blindness, a total of 442 subjects (148 males and 294 females, 13 to 55 years old, mean age of 27.06 years) participated in this experiment, including 245 students and 197 working individuals (details provided in Table 6). All participants provided informed consent prior to the experiment, and a symbolic reward was given upon completion of the study.

**Table 6:** Demographic information of participants.

Item		Student group	Working group
Gender	Male	76	72
	Female	169	125
Average age		22.79	32.37
Education	High school or below	5	13
	Associate degree	0	18
	Bachelor’s degree	76	85
	Master’s degree or above	164	81
Years of experience	Less than 3 years	N/A	66
	3–5 years	N/A	19
	5–10 years	N/A	59
	10–15 years	N/A	16
	More than 15 years	N/A	37

#### 4.2.2 Method

This experiment was conducted for 7 consecutive days (from Monday to Sunday) to further validate the measurement advantage of the HDRPS+ and the effect of continuous measurement. To minimize potential interference with participants’ normal work and life, participants were required to use a WeChat Mini-Program for HDRPS+ testing after completing their daily work.

**Figure 3:**
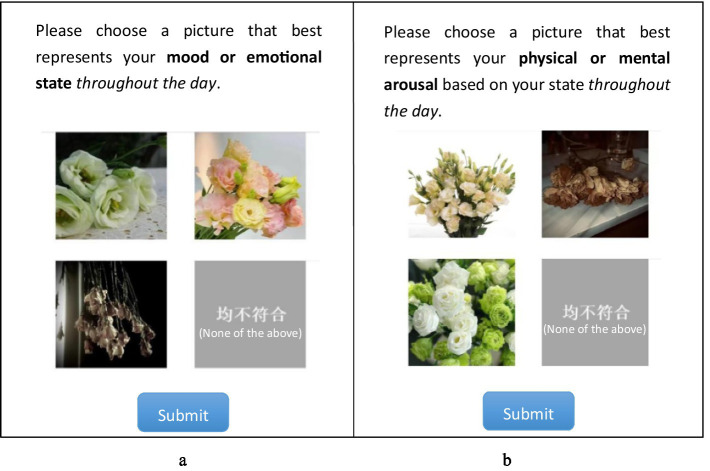
Example screenshots for **(a)** valence and **(b)** arousal tests.

The daily test consisted of three parts. First, HDRPS+ Test was administered with one type of picture (e.g., animals or plants or scenes) each day and required subjects to select 1 picture from 3 pictures (as well as an option of “none of the above”) with the same theme that best represented their valence or arousal level on that day, respectively (see Figure 3 for details). Meanwhile, since the pictures might induce emotional changes in the subjects (e.g., IAPS, NAPS, etc.), subjects were asked whether their affects were influenced by pictures after the HDRPS+ test was completed. The second is the SAM Scale Test, in which subjects were told to report their current valence and arousal levels using SAM scales. Third, after the daily test was completed, the mini-program provided feedback on the pictorial scale test results, and participants were asked to rate the accuracy of those responses using a 5-point scale. In addition, after the 7 daily tests were completed, subjects were required to complete the PANAS scale to assess their overall emotional states during the past week.

#### 4.2.3 Results

**Figure 4:**
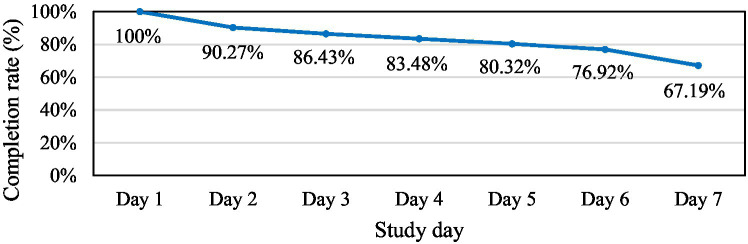
Completion rate of HDRPS+ over 7 days.

A total of 2,588 test sessions were completed by 442 participants. Daily logs showed the attrition inherent in continuous self-report: 80.32% of participants provided data on at least five workdays, and 67.19% completed every scheduled session (Figure 4). Laboratory research on pictorial questionnaires has shown higher response motivation than verbal formats (Baumgartner et al., 2019); our retention figures suggest that the pictorial HDRPS+ can likewise sustain participant engagement during field studies. However, it should be noted that prior studies that used realistic-picture emotion scales, including the original HDRPS (Liu et al., 2023), rarely report diary-retention figures, and because the present design combined our new pictorial tool with established scales, these completion rates are offered for descriptive context rather than as a basis for formal statistical comparison with traditional EMA or text-based surveys.

##### 4.2.3.1 Measurement mechanism test

To examine whether viewing the pictures altered participants’ affect, this study directly asked them to rate the influence of each set on a five-point scale (1 for “completely affected,” 2 for “greatly affected,” 3 for “moderately affected,” 4 for “slightly affected,” and 5 for “not affected at all”). Most respondents reported only slight or no change, and the ratings also clustered around the midpoint of the scale (M = 3.36, Mdn = 3, IQR = 2–4), suggesting that HDRPS+ can be administered without materially influencing the affect it is intended to measure.

##### 4.2.3.2 Reliability analysis

Reliability is pertained to the ability of an instrument to consistently measure an attribute (DeVon et al., 2007). Considering that affect is relatively short-lived affective states, traditional reliability testing methods (e.g., Cronbach’s alpha) are not applicable, we reviewed the development process of picture-based scales such as PAM and Affective Slider, and found that none of the prior studies had conducted reliability analyses due to the variability of affect.

Considering that reliability analysis is necessary for instrument development, this study used the results of the SAM test to calculate the consistency of the HDRPS+ measurement results, and used this as a reliability test result. The resulting consistency coefficients were 0.689 for valence (animals = 0.668, plants = 0.718, scenes = 0.666) and 0.645 for arousal (animals = 0.663, plants = 0.642, scenes = 0.631). All values exceed the 0.50 benchmark proposed by Cook et al. (2018), indicating that HDRPS^+^ demonstrates acceptable measurement stability across thematic categories.

##### 4.2.3.3 Validity analysis

Validity refers to the degree to which a tool measures what it intends to measure, and it can be divided into content validity, criterion-related validity, and construct validity (Lynn, 1986). As studies 1 and 2 have already ensured the content validity of the pictorial scale through rigorous experimental design, we mainly focus on assessing criterion-related validity and construct validity here.

Concurrent validity, as a criterion-related validity, refers to the degree of correlation between the results of a newly developed scale and an existing structurally similar scale, measured at the same point in time (DeVon et al., 2007). Generally, a coefficient greater than 0.45 has been commonly recommended as an indicator of concurrent validity (Cook et al., 2018). By calculating the correlation between HDRPS+ and SAM scale, we observed that the correlation of valence was 0.626**, correlation of arousal was 0.520**, both of which exceeded judgment criterion of concurrent validity, thus we can conclude that the HDRPS+ passed the concurrent validity test.

Construct validity can be examined through by convergent validity and discriminant validity. Convergent validity refers to the degree of similarity in measurement results when different measurement methods are used to measure the same characteristic, and 0.5 is generally used as an indicator of convergent validity (Cook et al., 2018). Since the HDRPS+ and SAM scale measure the same dimensions, the correlation coefficient between them can be used as the indicator of convergent validity judgment as well. Based on the criteria for evaluating convergent validity, we found that the HDRPS+ also passed the convergent validity test.

Discriminant validity refers to the degree of association between two theoretically unrelated constructs, with smaller correlation coefficients indicating better discriminant validity. Cook et al. (2018) suggested that a coefficient of less than 0.45 can be considered significant for discriminant validity. According to this, we calculated the correlation between the HDRPS+ and PANAS scales and found that all correlation coefficients were less than 0.45 (see Table 7 for details), indicating that the HDRPS+ is essentially different from the PANAS scale.

**Table 7:** Discriminant validity test.

Item	Arousal of the HDRPS+	PANAS	PA	NA
Valence of the HDRPS+	0.462**	0.314**	0.289**	−0.157**
Arousal of the HDRPS+	1	0.290**	0.249**	−0.163**

**p < 0.01.

##### 4.2.3.4 Analysis of social desirability effects

To gain a more intuitive understanding of the validity of the measurement results, we asked participants to provide direct evaluations (i.e., direct method) of the HDRPS+ measurement results using a 5-point scale after completing the daily test. The participants assigned values of 5, 4, 3, 2, and 1 to indicate “very accurate,” “accurate,” “average,” “not accurate,” and “not accurate at all,” respectively. The accuracy rate of HDRPS+ was found to be 78.03% based on this scoring method. We also used the SAM scale as a benchmark to calculate the accuracy rate of HDRPS+ (i.e., indirect method) and found that the accuracy rates of valence and arousal were 68.9 and 64.5%, respectively, which were lower than the accuracy rates obtained using the direct method.

Considering that the HDRPS+ was developed based on the technique of psychological projection and its measurement method is more concealed compared to the SAM scale, we have reason to believe that self-reporting using the SAM scale may lead to concealment behavior, whereas using the HDRPS+ for affective measures may mitigate the social desirability bias to some extent.

To further validate this inference, this study first collated and analyzed the distribution of the test results on the SAM scale and the HDRPS+ (see Table 8 for details), and revealed that subjects reported more positive and less negative emotions when using the SAM scale for valence measures. In terms of arousal distribution, subjects reported more high or more low arousal level when using the SAM scale. However, it is commonly assumed that individuals tend to have moderate arousal levels in their daily studies and work, which is more consistent with results obtained from the HDRPS+ test.

**Table 8:** Distribution of the test results.

Scale	Valence		Arousal	
	Category	Number	Category	Number
SAM	Positive	1,535	High	986
	Neutral	750	Medium	966
	Negative	303	Low	636
HDRPS+	Positive	1,203	High	933
	Neutral	985	Medium	1,124

## 5 Discussion

Affect measurement is one of the fundamental issues in the field of emotion science. Currently, in the field of organizational behavior, studies predominantly rely on discrete emotion theories, employing adjectives and recall-based measurement methods that fail to meet the demands for dynamic measurement. Moreover, existing dynamic methods face challenges such as high costs, contextual limitations, and constrained measurement results. This research is dedicated to the development and validation of a cutting-edge measurement tool, HDRPS+, which uses real images as anchors based on the theory of emotional dimensions, creating an intuitive, innovative, and fast tool applicable to organizational settings.

To address the limitations of HDRPS, the construction process was redesigned, involving experiments to evaluate benchmark images, validate effectiveness, assess emotional types, and evaluate emotional scores, ultimately creating a database containing 20 sets of theme-specific real images. Building on two-dimensional emotion theory, HDRPS+ was developed and tested with 85 participants to determine the optimal presentation method—displaying three images of the same category and theme at once. Subsequently, 442 participants were recruited for a seven-day diary study, which demonstrated that HDRPS+ possesses high reliability and validity, reduces the impact of social desirability, and achieves an accuracy rate of 78.03%.

In the duration of picture pool construction, we noticed that the contents of pictures with living subjects, such as animals and plants, are more likely to convey affective meanings, while pictures of non-living subjects, such as complex scenes, are difficult to express clear emotional meanings. Therefore, extra attention should be paid to the influence of vitality on emotional meanings when developing scales in the future. We also investigated the factors influencing the speed of the HDRPS+ test and found that female participants responded more slowly, possibly because women engage in a more elaborate cognitive-appraisal sequence when evaluating emotions (Lively, 2008). Furthermore, we analyzed the relationship between subjects’ affective state and test speed, and found that subjects in high-valence levels had the fastest testing speeds, which may be related to the attention bias moderated by affective proposed by Rozin and Royzman (2001) and Smith et al. (2003).

Upon analyzing the accuracy of HDRPS+, it was found that using HDRPS+ for valence testing could weaken the influence of masking behaviors to some extent, but arousal testing with this instrument reported more moderate arousal and less arousal fluctuations. Reviewing the previous studies, we suggest that HDRPS+ did not show the expected attenuating effect when measuring arousal, may due to problems in defining arousal (Schimmack and Grob, 2000). The SAM scale defines arousal as a process of change from relaxed and sleepy to excited and energetic, which implicitly includes two sub-dimensions: mental arousal and physical arousal. For mental arousal, individuals may experience a process of change from relaxed to tense, and they may report lower arousal to express a more relaxed personal state. For physical arousal, individuals may experience a process of change from sleepy and tired to energetic, and they may report higher arousal to present a positive and energetic personal state. Variations in the understanding of arousal contributed to the reporting of divergent arousal levels, indicating a need for a more nuanced approach in measuring arousal with HDRPS+.

This research features key innovations both theoretically and practically. First, it developed and optimized a cutting-edge research tool, verifying the image-emotion reflection mechanism and creating an emotional measurement tool that is intuitive, quick, and responsive. This represents a significant advancement in emotional measurement methods, providing valuable tools and data for studies in organizational behavior and emotion science. Second, in terms of application, HDRPS+ uses real images categorized by similarity to measure individual emotions through projective techniques directly, reducing the need for textual analysis and expression. This accelerates response times, decreases social desirability bias, and is particularly suited to the modern era’s reading and information processing demands, offering substantial practical value.

However, the study also presents certain limitations. Apart from the potential statistical bias that may result from our gender-imbalanced samples, several additional limitations deserve mention. First, although HDRPS+ separates valence and arousal with themed photographs, each assessment uses only three images per dimension, which may be too coarse to capture subtle affective nuances; future studies could compare HDRPS+ with slider-based tools such as the SAM grid or Affective Slider to achieve finer resolution. Second, the current picture bank contains only 20 theme-specific sets, so prolonged deployment (e.g., longer than 1 month) may introduce stimulus repetition and participant fatigue, highlighting the need to enlarge the image database. Third, although HDRPS+ appears to offer advantages like higher participant engagement and lower social-expectation bias, we could only assess these advantages preliminarily, as neither our study nor most prior work has collected comparable adherence data or explicit social-desirability measures. Future research should gather matched engagement data and include straightforward checks such as anonymous versus identified responding or physiological markers, to permit systematic comparisons. Finally, HDRPS+ was developed entirely within a Chinese context; despite a small validation with Chinese high-school students in the United States, its cross-cultural equivalence remains uncertain. Researchers applying HDRPS+ in other cultures should therefore conduct preliminary image evaluations and, if necessary, adapt the stimulus pool to the local context.

## 6 Conclusion

This study developed and validated HDRPS+, a novel emotional measurement tool based on the design principles of PAM and HDRPS, utilizing real images as anchors and suitable for organizational contexts. By verifying the image-emotion reflection mechanism, this research not only enhanced the intuitiveness and motivational appeal of the emotional measurement tool but also achieved rapid response judgment, demonstrating its innovative capabilities in the field of emotion measurement. Specifically, the application of HDRPS+ reduces the reliance on traditional textual analysis by directly measuring individual emotions through projective techniques, effectively minimizing social desirability biases and accelerating participant response times. These features make HDRPS+ particularly suitable for the digital age, aligning with modern needs for quick reading and information processing, and offering significant practical application value and broad developmental prospects. The study also revealed limitations of HDRPS+, including issues with emotional granularity, constraints in the diversity of image materials, and challenges in cross-cultural applications. Future efforts should focus on further optimizing the tool’s design and application to enhance its broad applicability and accuracy. The HDRPS+ tool, along with its criterion valence and arousal scores, can be requested at https://osf.io/d4wcn (Liu et al., 2024).

## Data Availability

The datasets presented in this study can be found in online repositories. The names of the repository/repositories and accession number(s) can be found at: https://osf.io/d4wcn.
