# Supplementary material for: HDRPS+: a new affective pictorial scale applicable to organizational contexts
Source: Front Psychol. 2025 Aug 8;16:1498143. doi: 10.3389/fpsyg.2025.1498143 (PMC12370707; doi:10.3389/fpsyg.2025.1498143)
Supplement: Supplementary file 1 [file Data_Sheet_1.pdf]

Appendix 1: Table I. Descriptive statistics of emotion pictures

Table I Descriptive statistics of emotion pictures

Group	Affective category distribution (Number)								Score distribution					
	Total	Positive	Neutral	Negative	Total	High	Medium	Low	V_{Mean}	V_{Min}	V_{Max}	A_{Mean}	A_{Min}	A_{Max}
Animals 1	9	4	2	3	9	5	1	3	3.25	1.67	4.57	3.26	1.90	4.19
Animals 2	8	2	2	4	8	4	1	3	2.85	1.96	4.31	2.96	1.94	4.45
Animals 3	9	3	2	4	8	4	1	3	3.10	1.91	4.67	3.31	1.97	4.5
Animals 4	7	3	2	2	7	3	1	3	3.37	2.16	4.49	3.13	1.86	4.38
Animals 5	8	2	2	4	8	4	1	3	2.87	2.11	4.22	3.11	1.88	4.14
Animals 6	7	3	1	3	8	6	1	1	3.17	2.21	4.29	3.55	2.14	4.12
Animals 7	5	1	2	2	9	6	1	2	2.85	2.37	3.58	3.52	2.05	4.26
Animals 8	3	1	2	0	6	4	1	1	3.57	3	4.14	3.66	1.98	4.45
Plants 1	9	4	1	4	7	2	1	4	3.08	1.60	4.74	2.63	1.67	3.67
Plants 2	9	5	1	3	9	4	1	4	3.29	1.34	4.55	2.82	1.33	4.09
Plants 3	9	4	1	4	9	4	1	4	2.82	1.05	4.47	2.83	1.24	4.24
Plants 4	9	3	2	4	7	2	1	4	2.74	1.53	4.17	2.57	1.66	3.88
Plants 5	9	4	1	4	8	3	1	4	2.8	1.16	4.52	2.64	1.33	3.93
Scenes 1	6	3	1	2	5	2	1	2	3.26	1.66	4.53	3.03	1.86	4.19
Scenes 2	8	2	2	4	8	3	1	4	2.58	1.12	4.16	2.80	1.78	4.09
Total	115	44	24	47	116	56	15	45	/	/	/	/	/	/

Appendix 2: Figure I: Examples of Different Presentation Methods

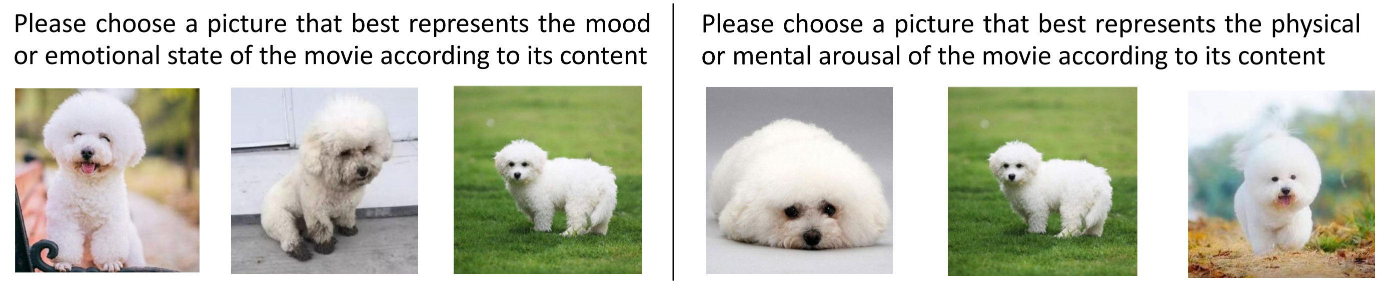

Figure I(a): Example of Primary Mode Selection for Valence and Arousal

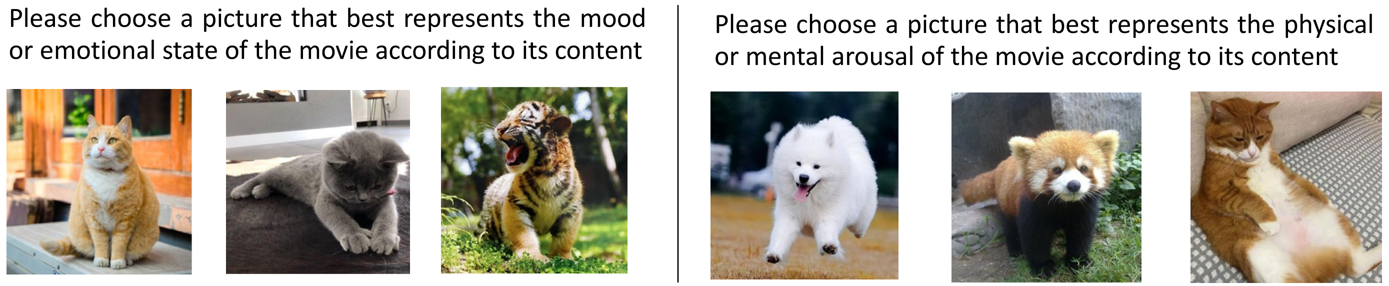

Figure I(b): Example of Middle Mode Selection for Valence and Arousal

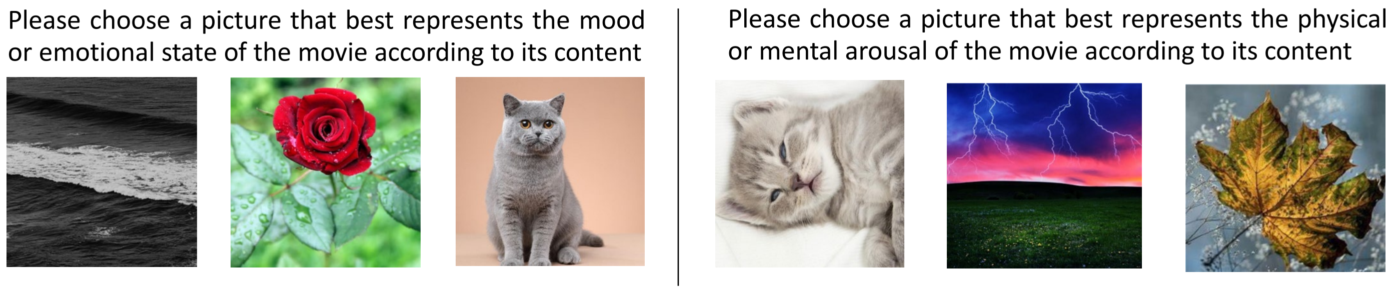

Figure I(c): Example of Advanced Mode Selection for Valence and Arousal
